# Policy Series: Illuminating the Intersection Between Employers and Experienced Employees: Current Research and Policy Directions

**DOI:** 10.1093/geroni/igaa057.2936

**Published:** 2020-12-16

**Authors:** Brian Kaskie

## Abstract

Population aging, resulting from the combination of longer life expectancy and lower birthrates, has widespread implications for both employees and employers across the United States. By2040, age55-plus workers are projected to account for more than25 percent of America’s workforce. Yet, even though we have gained a better understanding of the needs and preferences of aging workers, we know far less about the organizations which employ them. This symposium presents the latest research concerning: age discrimination in the workplace, intergenerational workplace arrangements, work ability and performance, and the increasingly varied pathways older persons are taking from work to retirement. We also examine how employers have addressed these issues, and consider why one type of employer may be more likely to adopt and implement a policy or program supporting older workers. Panelists then discuss policy alternatives that may increase and expand current employer efforts to support experienced employees.

